# Management of Iron Overload in Beta-Thalassemia Patients: Clinical Practice Update Based on Case Series

**DOI:** 10.3390/ijms21228771

**Published:** 2020-11-20

**Authors:** Valeria Maria Pinto, Gian Luca Forni

**Affiliations:** Centro della Microcitemia e delle Anemie Congenite Ente Ospedaliero Ospedali Galliera, Via Volta 6, 16128 Genoa, Italy; gianluca.forni@galliera.it

**Keywords:** thalassemia, iron overload, iron chelation therapy

## Abstract

Thalassemia syndromes are characterized by the inability to produce normal hemoglobin. Ineffective erythropoiesis and red cell transfusions are sources of excess iron that the human organism is unable to remove. Iron that is not saturated by transferrin is a toxic agent that, in transfusion-dependent patients, leads to death from iron-induced cardiomyopathy in the second decade of life. The availability of effective iron chelators, advances in the understanding of the mechanism of iron toxicity and overloading, and the availability of noninvasive methods to monitor iron loading and unloading in the liver, heart, and pancreas have all significantly increased the survival of patients with thalassemia. Prolonged exposure to iron toxicity is involved in the development of endocrinopathy, osteoporosis, cirrhosis, renal failure, and malignant transformation. Now that survival has been dramatically improved, the challenge of iron chelation therapy is to prevent complications. The time has come to consider that the primary goal of chelation therapy is to avoid 24-h exposure to toxic iron and maintain body iron levels within the normal range, avoiding possible chelation-related damage. It is very important to minimize irreversible organ damage to prevent malignant transformation before complications set in and make patients ineligible for current and future curative therapies. In this clinical case-based review, we highlight particular aspects of the management of iron overload in patients with beta-thalassemia syndromes, focusing on our own experience in treating such patients. We review the pathophysiology of iron overload and the different ways to assess, quantify, and monitor it. We also discuss chelation strategies that can be used with currently available chelators, balancing the need to keep non-transferrin-bound iron levels to a minimum (zero) 24 h a day, 7 days a week and the risk of over-chelation.

## 1. Introduction

Thalassemia syndromes are a group of inherited hemoglobin disorders including α-thalassemia, β-thalassemia, and E/β thalassemia. β-thalassemia is the most prevalent, with approximately 60,000 symptomatic individuals born annually worldwide [1,2]. Clinically, thalassemia is characterized by unbalanced globin-chain accumulation, ineffective erythropoiesis, chronic anemia, increased intestinal iron absorption, and consequent multi-morbidity [3,4]. Recent classification has distinguished thalassemic disorders into transfusion-dependent thalassemia (TDT; regular lifelong blood transfusions starting before the age of 2 years) and non-transfusion-dependent thalassemia (NTDT; occasional blood transfusions or limited periods of transfusion, such as for pregnancy or surgery) [5,6]. Transfusion requirements should be re-evaluated intermittently because patients can move from NTDT to TDT over time. The only current curative therapy is hematopoietic cell transplantation, but this is only available for a minority of patients [7]. Transfusion therapy can correct the anemia (the cause of death in TDT patients in the first decade of life) and significantly prolong survival [8,9]. The inability of the human organism to remove excess iron exposes TDT patients to its toxic effects. Consequently, patients no longer die from anemia, but die in the second decade of life from iron-induced cardiomyopathy [10,11].

The availability of iron chelators has dramatically improved survival by preventing and reversing heart failure [11]. Magnetic resonance imaging (MRI) [12,13,14,15,16,17,18,19], which can accurately monitor multiorgan iron overload (IOL), has enabled iron chelation therapy to be tailored to the individual patient’s needs. Regular transfusion therapy to maintain values of pre-transfusion hemoglobin (Hb) over 9 mg/dL reduces ineffective erythropoiesis [20], related bone dysmorphism, and splenomegaly, decreasing the need for splenectomy [21]. The improved quality and safety of transfused blood components [22,23] have reduced the risk of blood-borne viral infections. TDT patients can now survive into their 50 s and 60 s and enjoy a full life [11,21], and pregnancies are common [24]. Iron toxicity in TDT patients can affect all stages of development (erythropoiesis, growth, sexual maturation, endocrine homeostasis, cardiac, liver, and renal function, bone metabolism, aging) [25] and exposes them to an increased risk of malignant transformation [26,27,28,29,30]. In NTDT, even in the absence of regular red blood cell (RBC) transfusions, IOL occurs due to the enhanced intestinal absorption that is secondary to ineffective erythropoiesis and hepcidin suppression [31,32] and at slower rate than in TDT, making IOL a cumulative process with advancing age.

Due to differences in organ-specific iron transport, the rate of iron loading and unloading is much faster in the liver than in the heart and endocrine organs [12,13,33,34]. The aim of chelation is to consistently neutralize the toxic effects of iron and prevent or eradicate IOL [26,35,36,37,38], although the risk of over-chelation is a serious concern and the process requires management. In this case-based review, we discuss the general approach to iron chelation therapy (ICT) with currently available chelators in TDT and NTDT patients.

## 2. Pathophysiology of Iron Overload in Thalassemia

Iron homeostasis is a complex system that maintains daily absorption and excretion at approximately 2 mg/day. This is carefully regulated by several molecules [39]. Humans have no physiological mechanism to actively excrete iron, thus extra iron resulting from blood transfusion is stored in body tissues, leading to organ injury.

**Figure 1:**
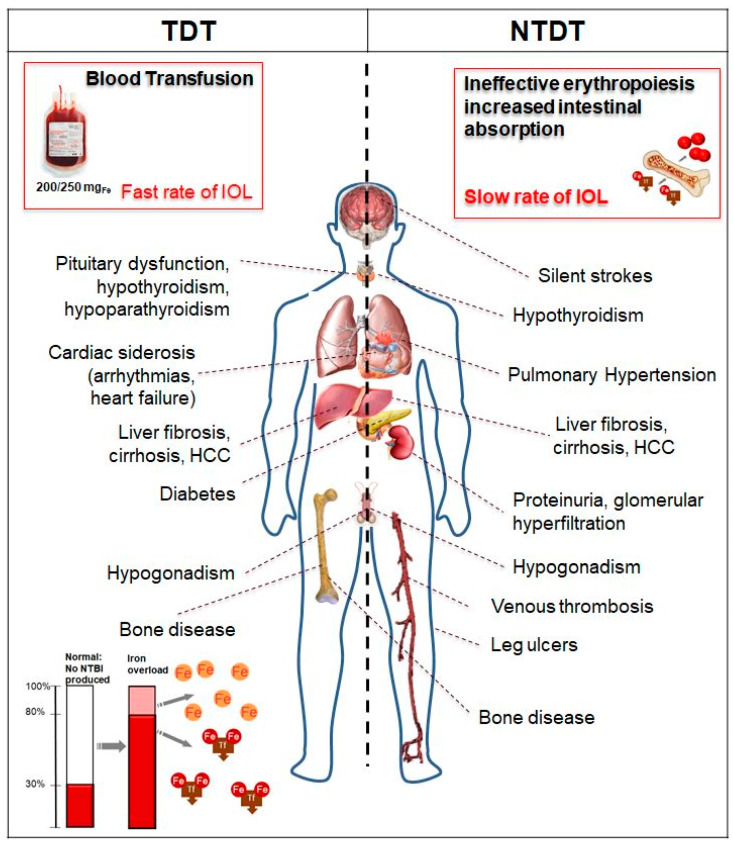
Target of iron overload (IOL) in transfusion-dependent thalassemia (TDT) and non-transfusion-dependent thalassemia (NTDT). HCC, hepatocellular carcinoma; NTBI, non-transferrin-bound iron; Fe, iron; Tf, transferrin.

Labile cellular iron (LCI), released after phagocytosis of transfused red blood cells by the reticuloendothelial system, binds to circulating plasma transferrin (two Fe^3+^ molecules). When the transferrin iron-binding ability is exceeded (transferrin saturation 60–80%), the non-transferrin-bound iron (NTBI) appears in the plasma and accumulates in different types of cells: hepatocytes, cardiomyocytes, and pituitary and pancreatic cells. In particular, a highly reactive Fe^2+^ subspecies of NTBI, labile plasma iron (LPI), can enter cells through calcium channels that are not regulated by intracellular iron concentration. Reactive oxygen species (ROS) produced by NTBI/LPI and LCI contribute to oxidant damage, cellular dysfunction, apoptosis, fibrosis, and necrosis in target organs, including the myocardium, liver, and endocrine glands. Iron transport through these channels is organ-specific and may explain the different loading rates observed by MRI [33]. Likewise, the rate of iron unloading in the liver is much faster than in the heart and endocrine organs [33]. Each unit of transfused packed RBCs (PRBCs) contains 200–250 mg of iron; therefore, 4800–12,000 mg of iron per year (2–4 PRBCs/month) is introduced in a usual transfusion regimen for a TDT patient, compared to 400–700 mg of iron absorbed from the diet per year, which is lost through cell sloughing and bleeding. In TDT, the predominant mechanism of IOL is secondary to transfusion therapy (Figure 1). In NTDT, IOL is a process that accumulates iron with advancing age, and it develops even in the absence of regular RBC transfusions. Secondary hepcidin suppression and enhanced intestinal absorption lead to preferential portal and subsequent hepatocyte iron loading and relatively lower levels of serum ferritin compared to TDT patients [31] (Figure 1). In NTDT, iron accumulation preferentially occurs in the liver rather than the myocardium [40,41].

### 2.1. Case 1: Severe Multiorgan Siderosis in TDT

AA, a North African female with transfusion-dependent beta (β)-thalassemia major, was referred to our center at 19 years of age. She has been transfusion-dependent since the age of 3 years, receiving 2–3 units of PRBCs/month, with a mean pre-transfusion hemoglobin of 7 g/dL. She underwent splenectomy at the age of 5 years, after which iron chelation was prescribed with subcutaneous deferoxamine (DFO) at 40 mg/kg 5 days/week. She presented normal growth and spontaneous menarche at 13 years. However, chelation therapy was stopped 5 years before referral to us because the drug was not available in her country of origin. Upon presentation at our center, her serum ferritin was 8760 μg/L and cardiac MRI T2* was 8.3 ms, indicating severe IOL with normal left ventricular ejection fraction (LVEF; >55%) confirmed by echocardiography. Electrocardiogram was normal. Her liver iron concentration (LIC) was 24 mg Fe/g dry weight (dw) tissue and pancreatic T2* was 8.1 ms. Liver ultrasound demonstrated hepatomegaly; liver enzymes were three times the upper normal value with negative serology for viral hepatitis. Pro-brain natriuretic peptide was normal. Her comorbidities included hypogonadotropic hypogonadism and impaired glucose tolerance (IGT), but there was no growth deficiency. We decided to treat the patient with combined therapy of deferiprone (DFP) at 90 mg/kg per day over three doses plus subcutaneous DFO at 50 mg/kg 12 h by micropump per day. We started weekly white blood cell counts, which is essential to monitor the risk of DFP-related agranulocytosis. After around 2 years, to prevent the risk of over-chelation and promote adherence, we stopped combined therapy and started deferasirox (DFX) 28 mg/kg per day in dispersible tablets. During the 2 years of combined therapy, we observed an increase in pancreatic T2* with regression of IGT. The patient has not experienced any therapy-related adverse events, and cardiac T2* continues to be >20 ms according to the latest imaging, with LIC < 5 mg Fe/g dw and abnormal pancreatic T2* (<20 ms). The patient’s serum ferritin, cardiac and pancreatic T2*, and LIC are reported in Table 1.

### 2.2. Case 2: ICT in TDT with Renal Function Alteration and Serum Ferritin < 500 μg/L

A 40-year-old Caucasian female with transfusion-dependent beta (β)-thalassemia major has been transfused from the age of 6 months, with a mean pre-transfusion hemoglobin of 9.8 g/dL. She was started on iron chelation with subcutaneous DFO 6 days/week at 3 years of age. Her parents were very compliant, with consequent good adherence to therapy for the patient. However, adolescence was characterized by long periods of discontinuation. The patient’s ferritin peaked at 2800 μg/L, and hypothyroidism and hypogonadism were observed. MRI data regarding IOL were not available at that time. At the age of 26, treatment was changed to DFX dispersible tablets, 30 mg/kg per day. Her mean transfusion iron intake was 0.4 mg/kg per day. Her first MRI measurement at the age of 26 showed an LIC of 8 mg/kg dw, a cardiac T2* of 23 ms, and a pancreatic T2* of 12 ms. At 39 years of age, she was apparently in good physical condition, and there was a persistent >33% increase in creatinine, a decrease in glomerular filtration rate (GFR), and a threefold increase in liver enzymes with negative serology for viral hepatitis (Table 2). The last LIC was 3 mg Fe/g dw, with median serum ferritin of 420 μg/L over the last two years, a cardiac T2* of 32 ms, normal LVEF, and a pancreatic T2* of 12 ms. Venous gas analysis was normal. She was taking DFX film-coated tablets, 14 mg/kg per day. The patient reported frequent self-administration of nonsteroidal anti-inflammatory drugs (NSAIDs) to control her back pain. She had severe osteoporosis (lumbar T-score –4.5). The patient stopped using NSAIDs, and creatinine, GFR, and liver enzymes returned to within the normal range without stopping chelation (Table 2). Therefore, we started to treat the patient with neridronate to address the osteoporosis; pain was significantly reduced [42].

### 2.3. Comments on Cases 1 and 2

In TDT patients, the most important and historical IOL complication is cardiac siderosis, which is responsible for heart failure and arrhythmias [11,14]. Thanks to advances in ICT, MRI monitoring of IOL, and overall disease management, mortality in TDT patients for cardiac disease has declined over time [19,43]. Specific cardiac MRI T2* thresholds have been associated with morbidity in TDT [44]. Patients with cardiac T2* > 20 ms (no detectable cardiac iron) do not typically develop heart dysfunction, while patients with T2* < 10 ms are at a proportionally higher risk of cardiac dysfunction and mortality [14,15].

Acute decompensated heart failure with a significant reduction in LVEF due to severe cardiac IOL (T2* < 10 ms) represents a medical emergency that requires treatment at a specialist center experienced in treating heart failure in thalassemia patients to improve cardiac outcomes [45,46]. Appropriate ICT should start as soon as possible to avoid life-threatening delays [45]. Toxic iron cardiomyopathy is reversible with the possibility of complete resolution of ventricular dysfunction by appropriate ICT. This is an important milestone achieved in the last few years in the management of thalassemic cardiac disease [45]. The three iron chelators, available in different parenteral and oral formulations, are currently used to treat IOL in patients with thalassemia: DFO by subcutaneous or intravenous injection, oral DFP tablet or solution, and DFX dispersible tablet for oral suspension or film-coated tablet (Table 3). DFO has the inconvenience of a parenteral route of administration (infused daily over 8–12 h, 5–7 days per week) and associated poor compliance, so oral agents such as DFP and DFX are generally preferred. Indeed, their introduction has improved compliance and has been associated with improved survival in patients with transfusion-dependent β-thalassemia [10,11]. All three chelators are effective in lowering NTBI/LPI and cardiac iron, but DFP appears to be more effective in cardiac iron clearance [47]. Combined DFP and DFO chelation therapy is superior to monotherapy in improving iron clearance, and this combination is the one most commonly used in cases of severe cardiac IOL [45]. Other combined regimens (DFO + DFX, DFX + DFP) [48,49,50] have demonstrated improvement of cardiac IOL and LVEF.

Case 1 developed severe multi-organ IOL with organ damage due to a lack of adequate ICT. LIC estimated from MRI, expressed in mg of iron per gram of liver dw tissue, correlates reliably with total body iron stores [51]. Normal LIC is <1.5 mg/g liver dw [35]. LIC values of >7 and >15 mg/g are usually used to indicate increased risk of complications and progressive liver fibrosis and cardiac mortality, respectively. However, in Case 1, the normal growth and spontaneous menarche showed that the patient had had good adherence to the therapy until around 14 years of age. LVEF was normal and IOL was due to the lack of availability of the chelator and not to poor adherence to the prescribed therapy. The lack of availability of the chelator highlighted the problem of providing regular ICT in settings with low and medium resources. We prescribed subcutaneous DFO by micropump and not intravenous administration to facilitate adherence. We knew from previous experience that the toxic iron effect on the heart could occur suddenly, and strict clinical follow-up and use of imaging techniques were maintained. Pre-transfusion hemoglobin was maintained at >9.5 g/dL with a mean transfusional iron intake of 0.5 mg/kg per day. The appropriate dose of an iron-chelating agent in relation to the rate of transfusional iron loading is an important factor in successful ICT [52,53]. The increase in liver enzymes observed in Cases 1 and 2 could have been related to viral infection, but that was excluded, and recent direct antiviral therapies have eradicated the transmission of hepatitis C virus (HCV) infection through transfusion, thus avoiding this confounding factor [54]. An increase in liver enzymes accompanied by abdominal pain can also be observed in decompensated heart failure due to liver stasis and Glissonian distension, and can be a cause of misdiagnosis in the emergency room [55,56]. However, in the cases under discussion, LVEF and pro-brain natriuretic peptide were normal, so increased transaminases were IOL-related in Case 1, and were due to NSAIDs in Case 2.

Thalassemia is associated with a markedly increased risk of endocrine disorders, hypogonadism, thyroid disorders, hypoparathyroidisms, impaired glucose metabolism, and osteoporosis [25,56,57,58,59]. The risk of endocrine complications is increased in patients showing poor compliance with iron chelation therapy. Endocrine complications were considered irreversible in the past. This concept has been changing since there is recent evidence showing that iron-related endocrine damage could be reversible or prevented with intensive ICT [36,58,60,61], as in glucose metabolism, but not as completely as in the heart. In Case 1, the IGT disappeared after ICT. The curve of unloading under intensive treatment in Case 1 highlights that the 50% rate of iron unloading in response to intensive ICT was over approximately 4–6 months for the LIC and approximately 14 months for the heart [12].

**Figure 2:**
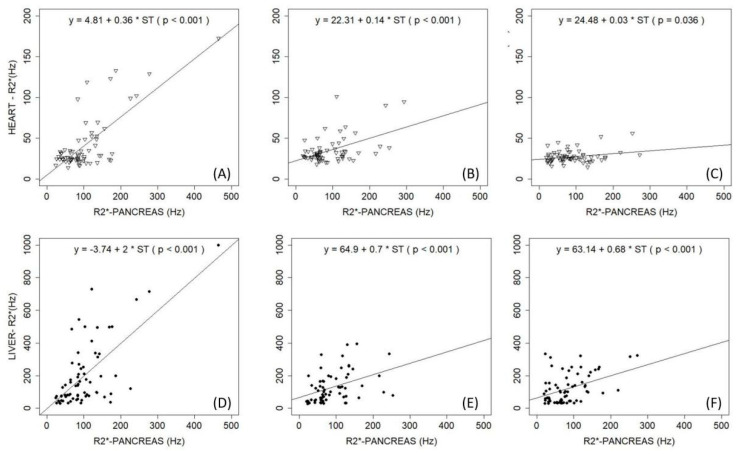
Observations at (**A**,**D**) baseline (time 0 years), (**B**,**E**) median value of 4 year follow-up, and (**C**,**F**) median value of 8 year follow-up. (**A**–**C**) Correlation of R2* values of pancreas and heart; (**D**–**F**) correlation of R2* values of pancreas and liver. With permission of Pinto et al. 2018 [62].

MRI assessment of the pancreas is difficult due to its small volume and tortuous course, its close proximity to air-filled bowel and stomach, and the frequent presence of fat involution, which causes oscillations in iron-mediated signal decay [16]. Removing iron from the pancreas seems to be more difficult compared to other organs. Pancreatic iron (evidence of a period of inadequate ICT) remains even after the liver and heart are unloaded [62] (Figure 2), as we observed in both Case 1 and In cases like these, with liver and heart unloaded, any attempt to remove the pancreatic overload by increasing ICT puts the patient at risk of over-chelation. Consequently, it is more important to consider the 24 h coverage effect of the chelators than the dose [35]. The back pain observed in Case 2 is a common concern in TDT, leading to the use or abuse of analgesic drugs, which may represent a confounding factor in ICT management because of the renal and liver toxicity from both drugs [25,42]. Despite optimized management, patients with β-thalassemia major continue to lose bone mass and develop osteoporosis and aging-related precocious sarcopenia [25,59,63]. The pathogenesis of thalassemia-associated osteoporosis is quite complex and multifactorial, involving IOL, nutritional and behavioral factors, endocrine and renal complications, and possibly also a genetic background [25,59,63,64]. Clinical data regarding the relationship between iron chelators and endocrine and bone disorders in thalassemia patients suggest a protective effect of DFX on bone mineral density, not described for the other iron chelators. DFX was also seen to produce a decrease in the prevalence of any endocrinopathy (−1.8%), which was not seen with other chelators [25].

In Case 2, renal involvement was observed; factors implicated in renal damage that are specific to thalassemia are anemia, IOL, and iron chelators [25].

Anemia can reduce systemic vascular resistance, leading to hyperdynamic circulation in glomeruli and a consequent increase in hydrostatic pressure within the glomerular capillaries. Anemia can also cause hypoxic damage that mainly affects cells in the proximal tubule because of their high rate of metabolic activity. This causes oxidative tubular injury, eventually leading to cellular apoptosis. Under anemic conditions, tubular cells can also change from an epithelial to a mesenchymal phenotype, with consequent tubular–interstitial fibrosis [25].

In addition to anemia, tubular oxidative stress in thalassemia can also be caused by iron, leading to tubular lipid peroxidation and subsequent cell injury/death. The tubular damage, which is probably dependent on the combined effects of IOL and hypoxia, is responsible for many urine abnormalities seen in thalassemic patients, such as increased proteinuria, albuminuria, calciuria, phosphaturia, uricosuria, and β_2_-microglobulin. Iron can cause kidney toxicity, but iron is an essential co-factor in renal prostaglandin synthesis, and consequently, a lack of prostaglandins due to iron depletion can reduce glomerular perfusion and GFR. Moreover, it is possible that the reduced GFR observed with DFO and DFX could be caused by so-called relative iron depletion.

The renal glomerular or tubular damage related to thalassemia can exacerbate the decline in renal function observed, even in healthy individuals, after the age of 40 years. GFR progressively decreases in about two-thirds of people, particularly men [25]. Although rare, acute renal failure caused by toxic tubular damage has been reported in patients receiving DFO. DFX can cause a generalized dysfunction of proximal tubular cells called Fanconi syndrome [65], characterized by hypokalemia, hypophosphatemia, hypercalciuria, metabolic acidosis, hyperaminoaciduria, and hyperuricosuria. In these rare cases, the drug should be withdrawn immediately. The most common renal adverse effect of iron chelators (mainly DFX) is a mild decrease in GFR, which is usually transient and reversible by tapering the drug dose [66]; sudden or aggressive iron removal could be related to excessive iron chelation as the cause of this hemodynamic effect [67]. Based on these observations, it is advisable to reduce the dose of iron chelators in patients who develop a significant decrease in renal function during treatment. A significant decrease in renal function is usually defined as a >33% increase in serum creatinine from baseline levels. It is particularly important to be aware of this hemodynamic effect in patients who may also have other contributing factors that reduce renal perfusion, such as fever or, as in Case 2, the use of NSAIDs.

Moreover, Case 2 emphasizes an emergent issue regarding the management of ICT when serum ferritin goes < 500 μg/L: authorized product information states that if this occurs, DFX must be interrupted. In our opinion, ICT should be continued even when ferritin is <500 μg/L and should not be completely stopped in order to maintain NTBI/LPI levels near zero, ensuring that a chelator is in the circulation at all times [35]. Administration of DFX at a mean dosage of 14 mg/kg per day is required to safely balance an iron intake of 0.3 mg/kg per day in the absence of IOL, with mild and transient creatinine and alanine aminotransferase fluctuations that do not require specific treatment [68]. In this setting of iron-chelated patients with low iron burden and “low” serum ferritin levels, close monitoring through liver and renal tests (Table 4) and an accurate examination of the patient’s history regarding any medicinal products they take are mandatory, considering that older patients are also more likely to develop age-related non-thalassemic conditions that could interact with the disease morbidity. As the median age of the thalassemia population increases, and considering that exposure to reactive iron is life-long, physicians must be aware of the potential for these patients to develop cancer [26,27,28,29,30].

The current literature suggests that the prevalence of cancers, particularly hepatocellular carcinoma and thyroid cancer, may be increased in patients with thalassemia compared with the general population [26,27,28,29,30].

Case 2 showed a change in renal and hepatic function that could be related to over-chelation; however, these changes were due to the use of NSAIDs.

### 2.4. Case 3: Liver Iron Overload in NTDT

A 61-year-old Mediterranean man with NTDT transfused only during splenectomy (performed at 33 years of age, 3 PRBC) was referred to our center from a peripheral center for asymptomatic liver test abnormalities consisting of mild elevation of serum transaminases (two times normal value) and elevated serum ferritin (1249 μg/L) and transferrin saturation (92%). Cholestatic enzymes were normal, mild hyperbilirubinemia was present (total bilirubin: 3.61 mg/dL; conjugated: 2.21 mg/dL). Hemoglobin was 9.8 g/dL, platelet count was 750.000/mmc. Coagulative tests, albumin, renal function, electrolytes, and metabolic parameters were normal. Ultrasound showed an enlarged liver with a nodular course, no focal lesions, and normal flow within the portal and hepatic veins. MRI showed a LIC of 12.7 mg Fe/g dw, indicating moderate to severe iron overload in the liver; cardiac MRI-T2* was >20 ms. Vibration-controlled transient elastography performed using a FibroScan^®^ device showed liver stiffness of 10.5 kilopascals, corresponding to a Metavir score of II. Hepatotropic viruses (HCV and hepatitis B virus, cytomegalovirus, Epstein–Barr virus) tested negative. Blood tests for autoimmune liver disease (anti-nuclear, anti-mitochondria, anti-liver-kidney microsome antibodies, anti-neutrophil cytoplasmic and anti-smooth muscle antibodies) were negative. The patient abstained from alcohol and his body mass index was normal. The patient started ICT with DFX film-coated tablets at a starting dose of 7 mg/kg per day, then adjusted to 10 mg/kg. LIC was 8.4 and 5.2 Fe/g dw at 6 and 12 months, respectively. Serum ferritin and transaminases decreased gradually. After 24 months, LIC was 2.8 Fe/g dw, serum ferritin was 320 μg/L, and ICT was stopped. The Fibroscan, at this time, showed the same Metavir score even though stiffness was reduced to 7.8 kilopascals. The patient has not experienced any adverse events related to therapy.

### 2.5. Comments on Case 3

The liver is the target organ of IOL in NTDT patients as result of the increased intestinal iron absorption that leads to preferential portal and hepatocyte iron loading. Iron in the liver is associated with an increased risk of hepatic failure resulting from hepatic fibrosis and cirrhosis. These complications are the result of damage caused not only by ROS but also by the profibrogenic effect of iron [69,70].

The time factor is important because the longer duration of hepatic iron exposure in NTDT patients explains why morbidity is mainly evident in older adults [71,72]. Mortality due to liver disease accounts for 10% among NTDT patients [73].

DFX is the only iron chelator specifically approved for NTDT patients 10 years and older when deferoxamine therapy is contraindicated or inadequate [3]. Data from trials applying MRI technology have shown that all three chelators are very effective and safe at removing iron from the liver [47,60,74,75,76,77], although there is consolidated data on DFX showing efficacy in reducing severe iron overload, and it has demonstrated the ability to reduce hepatic fibrosis and inflammation [78]. It is important to underline that liver enzymes can be increased with LIC >3 mg/g dw, therefore transaminase values of 3 to 5 times the norm should not prevent the starting of chelation in this case [36]. With LIC < 3 mg/g dw, increased transaminases should draw our attention to the risk of overchelation, which requires prompt interruption of ICT [36].

## 3. Diagnosis and Monitoring Iron Overload

Table 5 summarizes the methods for clinical diagnosis and monitoring of IOL [4,17,38,79,80,81,82,83].

MRI is increasingly used to diagnose and monitor iron concentrations in the liver and heart for its reliability and safety [17,38,44,83,84]. Iron shortens the T2 and T2* relaxation times measured by MRI, and it was demonstrated that their reciprocals, R2 and R2*, are directly proportional to iron concentration [17]. LIC can be used to estimate total body iron [51]. IOL evaluations are usually performed with 1.5-Tesla MRI scanners. MRI offers the advantage of inter- and intra-scanner reproducibility, correlates with cardiac function, and relates to tissue iron concentrations. The 3-Tesla MRI scanner is also indicated when there are low levels of iron, such as in brain and pituitary; for the liver, heart, and pancreas, 1.5-Tesla scanners are considered to be better and do not have the artifacts that affect 3-Tesla scanner images, especially for high iron levels [17,18]. The small dimensions of the pancreas, its tortuous course, its position within the body, and possible fat involution make it more difficult to assess IOL in this organ than in the liver with MRI.

Pituitary and renal iron can also be measured by MRI, but they are not standard or routinely available [18].

Serum ferritin remains a widely used marker to assess IOL. Yearly trends are a good indication of systemic IOL status [5,85]. In NTDT, serum ferritin level correlates with IOL [86]. Trends in ferritin levels should be examined for therapeutic decision-making where MRI is not available [87]. Transferrin saturation is an indirect measurement of the NTBI/LPI pool.

## 4. Iron Toxicity and Chelation

Intensive ICT was seen to successfully restore highly compromised heart function (LVEF from >20 to <55) in a few weeks, much faster than cardiac iron unloading, confirming the importance of removing the NTIBI/LPI toxic effect. Iron-related multi-organ damage in minimally chelation adherent TDT starts discreetly from the liver to the endocrine glands in infancy and childhood, and later goes on to involve the pancreas and heart [38]. Concomitant factors such as HCV infection result in a striking increase in the risk of tissue damage [88]. In this way, and as seen in the intriguing Coates empiric equation, total iron toxicity is related to the sum of exposure over time to the reactive iron, modulated by environmental and genetic antioxidant factors [33].

The goal of IOL treatment is to achieve a neutral or negative iron balance, reducing plasma and cytosolic levels of reactive low-molecular-weight “labile” iron pools that are constantly being generated (Fe^2+^; NTBI/LPI) to zero and therefore preventing iron damage to tissue [26,35,36,37,38]. So, the efficiency of chelation depends on iron chelators being available 24 h a day.

The main characteristics of iron chelators are summarized in Table 3 [53,89,90]. Indications and approval status vary across countries [91,92,93,94].

As we mentioned, data from trials applying MRI technology [47,60,74,75,76,77] have shown that all three chelators are effective at lowering NTBI/LPI and are safe and very effective at removing iron from the liver and heart. ICT should be tailored according to the patient’s IOL profile (side effects, adherence, transfusion iron intake, etc.). Appropriate dose changes during the course of ICT are crucial for a successful outcome [52,53]. Chelation intensity should be increased in the presence of multiorgan IOL and signs of organ damage and reduced to the lowest dose in patients experiencing successful unloading of iron, in order to avoid toxic effects of chelation rather than toxic effects of iron. Renal function decreases with advancing age, as described above, so in older well-chelated patients we use a lower dose, such as in low-risk patients with myelodysplastic syndrome [95]. Different combinations of iron chelators, including DFO + DFP, DFO + DFX, and DFP + DFX, are also used in clinical practice [60,96,97,98]. We alternate between DFP and DFX every day with good results in those rare patients who are not able to continue daily use of a single chelator [99].

Chelation should start after 2 years of age once the patient has received 10 transfusions, with ferritin 1000 μg/L or LIC > 3 mg/g dw. However, LIC is not usually available for babies or infants due to the challenges of MRI image acquisition in these young patients. We sometimes start chelation with low-dose DFX before 2 years of age with close monitoring of motor and cognitive development, with the aim of preventing serious IOL and prolonged exposure to iron’s toxic effects, because, as described above, any endocrine overload is only partially reversible [36]. Nevertheless, discontinuing ICT because of teratogenic considerations should be recommended during pregnancy. We restart ICT with DFO after the first trimester, given the increased blood consumption during pregnancy. We recommend assessment of LIC, cardiac T2*, and cardiac function as part of family planning to ensure a lower iron burden before conception.

In transplant thalassemia patients, phlebotomy is the gold standard to reduce iron burden and return body iron to within the normal range. DFO is a proven alternative; in this situation, we also use low-dose DFX [100].

In NTDT, the less severe but longer duration of iron exposure explains why morbidity is mainly evident in older adults. In NTDT, iron exposure is associated with hepatic fibrosis, risk of hepatocellular carcinoma, risk of developing thrombosis, pulmonary hypertension, silent stroke, hypothyroidism, hypogonadism, osteoporosis, and renal disease [101,102,103,104]. In NTDT patients, the indication to start DFX or DFO therapy (DFP is off-label use) is LIC ≥ 5 mg/g dw in those >10 years of age. Chelation should be interrupted if LIC < 3 mg/g dw is observed. Alternatively, serum ferritin can be used to indicate the need to initiate (>800 μg/L) or interrupt (<300 μg/L) iron chelation [86,103,105]. The risk is to chelate too late, when irreversible complications have already occurred, complications which can themselves compromise chelation therapy. We use a starting dose of DFX 7 mg/kg per day when ferritin is >350 μg/L [106] and transferrin saturation is >70% and LIC > 3 mg/g dw. However, a consolidated and validated strategy to treat NTDT patients is an unmet medical need.

Moreover, successful IOL management depends on long-term adherence to ICT [107,108,109,110]. Poor adherence to the prescribed therapy is the main cause of treatment failure [110]. Barriers to optimal adherence are related to practical factors such as intolerance to a chelator (drug-related side effects; i.e., gastrointestinal tolerability), preparation time, palatability, difficulty with DFO infusion, or psychological and psychosocial factors. Adolescence and the transition to adult care is a critical time for patients and caregivers to maintain optimal adherence. Every effort should be made by all members of care centers to support patients and caregivers in understanding the importance of adhering to therapy by making information available and raising awareness, providing age-appropriate education. Psychosocial support throughout the lifespan should be part of standard care in the management of thalassemia, a chronic disease, with special emphasis on the multidisciplinary team approach, which include physicians, nurses, psychologists, and social workers.

## 5. Conclusions

Advances in monitoring iron burden and improved ICT and global management of thalassemia are improving the prognosis and survival of patients worldwide. However, IOL remains an important issue in these patients, accounting for most complications and cardiac disease, particularly in low-resource settings. Avoiding iron damage means offering thalassemia patients a chance for curative therapy in the future [111].

The key points in IOL management are as follows: (i) encourage long-term patient adherence through patient education, shared decision making, pharmacist support, and motivational interviewing; (ii) conduct adequate assessment and monitoring of ICT with the aim of removing iron toxicity 24/7 and maintaining body iron levels near normal and safe ranges; (iii) avoid the risk of over-chelation; and (iv) monitor cancer risk in adult patients.

## Figures and Tables

**Table 1:** Case 1: Characteristics of 19-year-old patient with transfusion-dependent β-thalassemia major with severe multiorgan iron overload (2015–2018).

	Target	2015	2016	2017	2018
Pre-transfusion hemoglobin (g/dL)	>9.5	9.5	9.6	9.8	9.7
Ferritin * (μg/L)	<200	8760	2450	864	451
Transaminases *	NV	3 × ULN	NV	NV	NV
LIC (mg/g dw)	<1.5	24	9	4	3.8
MRI-T2* Heart (ms)	>20	8.3	16	24	26
LVEF (%)	>55	>55	>55	>55	>55
MRI-T2* Pancreas (ms)	>20	8.1	12.1	16	21
ICT		DFP (90 mg/kg) DFO (50 mg/kg)	DFP (90 mg/kg) DFO (50 mg/kg)	DFX dispersible tablet (28 mg/kg)	DFX film coated tablet (14 mg/kg)
Iron intake (mg/kg/day)	0.3–0.6	0.5	0.5	0.5	0.5

* Annual average. NV, normal value; ULN, upper limit of normal; LIC, liver iron concentration (measured in dry weight tissue); MRI, magnetic resonance imaging; LVEF, left ventricular ejection fraction (by MRI); ICT, iron chelation therapy; DFP, deferiprone; DFO, deferoxamine; DFX, deferasirox.

**Table 2:** Case 2: 40-year-old patient with transfusion-dependent β-thalassemia major with renal function alteration and serum ferritin <500 μg/L.

	Target	T_0_	After 3 Months	After 6 Months
Pre-transfusion hemoglobin (g/dL)	>9.5	9.8	9.8	9.7
Ferritin (μg/L)	<200	420 *	415	402
Transaminases	NV	3 × ULN	NV	NV
Creatinine (mg/dL)	<1.1	1.2	0.8	0.7
CrCl (mL/min)	>60	54	81	92
Iron intake (mg/kg/day)	0.3–0.6	0.4	0.4	0.4
ICT		DFX film-coated tablet (14 mg/kg)	DFX film-coated tablet (14 mg/kg)	DFX film-coated tablet (14 mg/kg)
NSAIDs		Yes	No	No

* Two-year average. T0, baseline; NV, normal value; ULN, upper limit of normal; CrCl, creatinine clearance (Cockcroft–Gault method); ICT, iron chelation therapy; DFX, deferasirox; NSAIDs, nonsteroidal anti-inflammatory drugs.

**Table 3:** Main characteristics of iron chelators.

Chelator	DFO	DFP	DFX
Structure	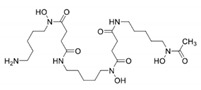	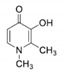	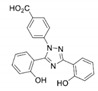
Molecular weight	560	139	373
First clinically available	1968	1999	2005
Administration route	Parental (subcutaneous or intravenous)	Oral (tablets or solution)	Oral (dispersible or film-coated tablets)
Administration frequency	8–12 h, 5–7 days per week; continuous infusion over 24 h in heart failure	Every 8 h, TID	Once daily, ongoing evaluations on BID dosing
Plasma half-life	30 min	3 h	8–16 h
Route of iron excretion	Urinary and fecal	Urinary	Fecal
Recommended dose	30–60 mg/g per day	75–100 mg/kg per day	20–40 mg/kg per day (dispersible tablets) or 14–28 mg/kg per day (film-coated tablets)
Main adverse event	Reaction at site of infusion, severe allergic reactions, bone abnormalities, growth failure, auditory (hearing loss), ophthalmologic (retinal damage), Yersinia infection	Gastrointestinal, arthralgia, transient increase in liver enzymes, neutropenia, agranulocytosis	Increased GFR and serum creatinine, proteinuria, rare renal failure, increased liver enzymes, rare liver failure, skin rash, gastrointestinal, rare gastrointestinal bleeding
Pregnancy	Contraindicated (can be used only at the end of the second trimester in patients with severe heart and liver IOL)	Contraindicated	Contraindicated
Licensed use—TDT	Treatment of chronic IOL resulting from transfusion-dependent anemia	Treatment of transfusional IOL in TDT where DFO is contraindicated or inadequate	US: Treatment of transfusional iron overload in patients 2 years or older Europe: Treatment of transfusional iron overload in patients 6 years and older, and when DFO is contraindicated or inadequate, in patients 2–5 years old
Licensed use—NTDT	No sufficient data, commonly used in clinical practice	Off-label	US: Treatment of chronic iron overload in patients 10 years of age and older with LIC ≥5 mg/g dry weight liver and SF ≥300 μg/L Europe: Treatment of chronic iron overload in patients 10 years of age and older with LIC ≥5 mg/g dry weight liver and/or SF ≥800 μg/L
Cost/year (£)	5584	5519	23,179

DFO, deferoxamine; DFP, deferiprone; DFX, deferasirox; TID, three times daily; BID, twice daily; GFR, glomerular filtration rate; IOL, iron over load; TDT, transfusion dependent thalassemia; IOL, iron overload; ICT, iron chelation.

**Table 4:** Scheme of nephrology monitoring examinations.

Tests	Baseline	1st Month	6th Month	Every 6 Months
Nephrology visit	X			
General functional indices				
Creatinine	X	X	X	X
Urine test	X	X	X	X
Cystatin C	X	X *	X	X
Proteinuria/creatininuria (mg/g)	X		X **	X
Tubular functional indices				
β2-microglobulin/creatininuria (μg/g)	X			X
Calciuria/creatininuria (mg/g)	X			X
Phosphaturia/creatininuria (mg/g)	X			X
NGAL/creatininuria (μg/g)	X			X
Venous blood gas analysis	X			X ***
Glomerular functional indices				
Albuminuria/creatininuria (mg/g)	X		X **	X

NGAL, urine neutrophil gelatinase-associated lipocalin. X indicates checkup. * Every week for 1 month only in patients treated with DFX. ** Every month for the first 6 months only for patients treated with DFX. *** For the first 3 years. Modified from Pinto et al. 2019 with permission [25].

**Table 5:** Quantification of iron overload: methods [4,17,38,79,80,81,82,83].

Method	Advantages	Limitations
Magnetic resonance imaging (MRI)	− Noninvasive − Safe − Considered the standard of care − Reciprocal of T2/T2* linearly related to LIC − Reliable precision and accuracy based on standardized validation procedures − Measurement of morphological and functional parameters − Widely used worldwide − Inter-scanner reproducibility − Intra-scanner reproducibility − Multi-organ evaluation	− Indirect measurement of LIC − Need for calibration to convert measured T2/T2* into iron concentration − Calibration is organ-specific − Patients with ferromagnetic inserts in their tissues cannot undergo examination − Trained personnel required for acquisition and post-processing − Costly − MRI scanners not always available
	Timing: − LIC: Q 2 years if <3 mg/g; Q 1 year if 3–15 mg/g; Q 6 months if >15 mg/g or rapidly increasing trend in serum ferritin/LIC − Cardiac T2*: Q 2 years if ≥30 ms; Q 1 year if ≥10 to <30 ms; Q 6 months if <10 ms	
Serum ferritin	− Inexpensive and easy to use for repeated assessment and measurement − Possible to identify trends with repeat samples − Correlates with both total body iron stores and clinical outcomes (high levels of iron overload imply high levels of serum ferritin); most common method to monitor ICT and only method available for several centers	− Indirect estimate of iron burden − Nonlinear response to iron load at high levels − No decrease does not exclude response − Non-Fe-related conditions (infection, inflammation, liver disease) may influence serum ferritin levels
	Timing: − Q 3–4 months (exclude ongoing infections or inflammation at time of measurement)	
Transferrin saturation	− Inexpensive and easy to assess − Only commonly used test that reflects toxic NTBI/LPI pool − Over 70% means NTBI/LPI are definitely significantly increased − Indirect measurement of NTBI/LPI pool	− Not reliable for monitoring poly-transfused patients
NTBI/LPI	− Corresponds to potentially toxic form of circulating iron − Normalizing NTBI/LPI is an important goal for potentially toxic form of circulating iron	− Not yet a routine test − Highly labile − Rapid return or even rebound after an iron chelator has been cleared − Complexity of interpreting levels affected by other factors (ineffective erythropoiesis, phase of transfusion cycle, rate of blood transfusion)
Liver biopsy	− Direct quantification of iron overload − Evaluation of liver histology that cannot yet be reliably estimated by noninvasive methods − Widespread use before introduction of noninvasive techniques made it the gold standard for LIC measurement − Still the only possible way to directly measure LIC for centers without 1.5 Tesla MRI scanners	− Invasive method − Risk of complications − Poorly accepted by patients − Inadequate sample size or uneven distribution of iron (i.e., in presence of cirrhosis) could provide misleading results − Local measurement of iron overload − Invasive nature makes it impossible for therapeutic follow-up
Biosusceptometry	− Noninvasive − High sensitivity − Use of low magnetic field	− Not widely available/used − Limited to liver

ICT, iron chelation therapy; NTBI, non-transferrin-bound iron; LPI, labile plasma iron; LIC, liver iron concentration; MRI: magnetic resonance imaging; Q, every.

## Abbreviations

TDT	Transfusion-dependent thalassemia
NTDT	Non-transfusion-dependent thalassemia
MRI	Magnetic resonance imaging
IOL	Iron overload
Hb	Hemoglobin
RBC	Red blood cell
LCI	Labile cellular iron
NBTI	Non-transferrin-bound iron
LPI	Labile plasma iron
ROS	Reactive oxygen species
PRBC	Packed red blood cell
DFO	Deferoxamine
LVEF	Left ventricular ejection fraction
LIC	Liver iron concentration
Dw	Dry weight
IGT	Impaired glucose tolerance
DFP	Deferiprone
DFX	Deferasirox
GFR	Glomerular filtration rate
NSAIDs	Nonsteroidal anti-inflammatory drugs
ICT	Iron chelation therapy
HCV	Hepatitis C virus
